# Development of Complex Models to Study Co- and Polymicrobial Infections and Diseases

**DOI:** 10.1371/journal.ppat.1005858

**Published:** 2016-09-08

**Authors:** Glenn Rall, Laura J. Knoll

**Affiliations:** 1 Fox Chase Cancer Center, Philadelphia, Pennsylvania, United States of America; 2 Department of Medical Microbiology and Immunology, University of Wisconsin–Madison, Madison, Wisconsin, United States of America

There is an inherent catch-22 in performing research that aims to better understand a clinical disease: to make true strides, one needs to simplify the system sufficiently to test individual variables. However, reducing a complex process to an experimentally approachable, simplified system may be so reductionist that the results are irrelevant to the disease in question. For many of us, animal models have proven to be remarkably powerful. Indeed, many biological principles were uncovered and major clinical advances made using animal models, and the advent of transgenic, knockout, and conditional animals has further cemented their value to the pathogenesis field. While animal models of infection have been invaluable, the simplified nature of most models has prevented a complete understanding of the more complex ways by which pathogens can cause human disease. For example, much of what we know about infectious agent pathogenesis has been gained from the use of adult, immunocompetent, immunologically naïve, inbred mice infected with a single pathogen. In stark contrast, humans with vastly different immunological histories and genetic backgrounds are colonized by a diverse array of microbial communities and likely bombarded with temporally overlapping antigenic stimuli. Despite this discrepancy between mouse models and the human experience, we know very little about how concurrent immune responses interact and contribute to disease in either humans or mice. This gap in knowledge is important to remedy because many human diseases are caused by polymicrobial exposures—including pneumonia, peritonitis, and others (e.g., hepatitis and Lyme’s disease [1,2])—that can have exacerbated symptoms when combined with a second pathogen. Moreover, any immunogen, even those that are noninfectious, may be relevant in this context, including allergens and vaccinations or preexisting chronic conditions such as cancer or autoimmunity. Many who have begun to explore this area in more depth have contended that temporally overlapping infections or immune responses may play a foundational role in some poorly understood inflammatory illnesses in humans.

If models are to be useful to fully explore this hypothesis, then more complex systems must be developed. Guiding the development of such systems should be fundamental questions such as: Is the immune response to a given immune stimulus functionally identical, regardless of whether the host is challenged with other, distinct immunogens? How might the response to one challenge influence the induction or trafficking of lymphocytes to a different challenge, especially if the two immunogens are tissue-restricted? Most critically: how might temporally overlapping infections alter pathogenesis? That is, could novel diseases occur only following simultaneous immunogenic encounters? One can envision that coincident infections could change a pathogenic outcome either directly or indirectly. Direct pathogen–pathogen interactions can exacerbate disease, as seen in otitis media, pneumonia, and sinusitis [1,3]. Alternatively, activated immune responses may interact in various ways to provoke illness. These include: (i) immunological interference, in which concurrent Th1 and Th2 immune responses limit efficient pathogen clearance [4–6]; (ii) cross-reactive responses, in which epitopes from otherwise diverse pathogens may be recognized by the same T cell receptors [7,8]; and (iii) persistent infections, such as those caused by some viruses, bacteria, or parasites, which confer protection to a subsequent acute challenge by resetting the homeostatic immunological “setpoint.”

To incentivize more thinking in this emerging scientific area, we have selected research articles from the *PLOS Pathogens* archive that directly tackle some of these issues. Of the over 70 excellent articles that the journal has published to date, we have chosen a select few from a diversity of systems that we believe are of particular interest to feature in the new *PLOS Pathogens* collection, “Bridging Communities: Co- and Polymicrobial Infections.” These articles have made substantive mechanistic advances to reveal how overlapping infections, or the immune responses they stimulate, might interact in novel ways. With this subset, we have also endeavored to underscore some of the unique approaches used by investigators in the various pathogenic disciplines *PLOS Pathogens* supports. Many more published articles that cover this area are available in the Supporting Information (S1 Table).

More than just a historical appreciation, we hope that this collection will promote deeper discussions and increased research article submissions focused on polymicrobial infections, especially by trainees who are considering what fields to pursue as independent investigators. In addition to the importance of the science that is done, research on polymicrobial infections and disease will bridge scientific communities, break down walls that preclude truly free exchanges of ideas and reagents, and expedite discovery of new principles. We anxiously look forward to the surprising and important insights such efforts will yield.

## Supporting Information

S1 Table Additional *PLOS Pathogens* co- and polymicrobial published articles. (XLSX)
